# Pitch-Derived Activated Carbon Fibers for Emission Control of Low-Concentration Hydrocarbon

**DOI:** 10.3390/nano9091313

**Published:** 2019-09-14

**Authors:** Hye-Min Lee, Byeong-Hoon Lee, Soo-Jin Park, Kay-Hyeok An, Byung-Joo Kim

**Affiliations:** 1 Research Center for Carbon Convergence Materials, Korea Institute of Carbon Convergence Technology, Jeonju 54852, Korea; 2 Department of Chemistry, Inha University, Incheon 22212, Korea; 3 Department of Chemical Engineering, Chonbuk National University, Jeonju 54896, Korea; 4 Department of Nano & Advanced Materials Engineering, Jeonju University, Jeonju 55069, Korea

**Keywords:** pitch, activated carbon fiber, evaporated fuel, hydrocarbon emissions

## Abstract

The unburned hydrocarbon (HC) emissions of automobiles are subject to strong regulations because they are known to be converted into fine dust, ozone, and photochemical smog. Pitch-based activated carbon fibers (ACF) prepared by steam activation can be a good solution for HC removal. The structural characteristics of ACF were observed using X-ray diffraction. The pore characteristics were investigated using N_2_/77K adsorption isotherms. The butane working capacity (BWC) was determined according to ASTM D5228. From the results, the specific surface area and total pore volume of the ACF were determined to be 840–2630 m^2^/g and 0.33–1.34 cm^3^/g, respectively. The butane activity and butane retentivity of the ACF increased with increasing activation time and were observed to range between 15.78–57.33% and 4.19–11.47%, respectively. This indicates that n-butane adsorption capacity could be a function not only of the specific surface area or total pore volume but also of the sub-mesopore volume fraction in the range of 2.0–2.5 nm of adsorbents. The ACF exhibit enhanced BWC, and especially adsorption velocity, compared to commercial products (granules and pellets), with lower concentrations of n-butane due to a uniformly well-developed pore structure open directly to the outer surface.

## 1. Introduction

As the interest in environmental pollution increases, regulations of automotive emissions are being made stronger around the world [1,2]. Automotive emissions are divided into evaporation gas and exhaust gas. The exhaust gas is generated by the combustion of fuel and consists of harmful substances such as CO*_x_*, NO*_x_*, SO*_x_*, and particulate matter (PM) [3,4,5]. The evaporation of unburned fuel from the fuel tank generally forms evaporation gas [6]. Its main component is hydrocarbon (HC) molecules, and there are many studies that indicate that HC is one of the critical materials causing smog, mist, and lung cancer [7,8].

Evaporation gas is normally generated even when the car is parked or being refueled; thus, it is difficult to prevent this using a separate actuator [9]. The evaporation gas is systematically collected and concentrated by means of fuel-vapor-emission control systems (a carbon canister and hydrocarbon trap sheet composed of activated carbon pellets and an activated carbon sheet, respectively; ACP and ACS) [10,11,12]. When the engine starts, the collected evaporation gas is transferred with air to the engine and used to enhance the fuel efficiency [13,14].

The diurnal regulation value for unburned HC emissions in cars was previously 500 mg/test (LEV-II), but recently it was further strengthened to 300 mg/test (LEV-III) [2]. Especially, canister bleed emission limits have been set at 20 mg/test for passenger car [2]. In the past, fuel-vapor-emission control systems were studied to enhance the pore characteristics of activated carbon or to increase the apparent density in order to adsorb larger amounts of evaporation gas [15,16,17]. However, for the fuel-vapor-emission control systems that meet the new stringent regulations (LEV-III), it is necessary to develop activated carbon capable of adsorbing a larger amount of evaporation gas and of completely adsorbing low concentrations of evaporation gas at the same time [2]. In order to solve this problem, a method for applying a material for the selective adsorption of low-concentration evaporation gas by an existing carbon canister, and a method for adding a separate device such as a ceramic honeycomb, have been discussed [10].

Activated carbon (AC) occurs in shapes classified as granules [18], pellets [19], and fibers [20,21,22]. Granular and pellet AC have a higher bulk density than fibrous activated carbon (activated carbon fiber, ACF), and so they can absorb a larger amount of harmful substances because of the larger input mass with the same volume [16,23]. Pellet AC usually exhibits lower pressure drop performance compared to granular AC [24,25], and so it is widely used in adsorption towers, canisters, and air cleaners. The ACF has a faster adsorption rate and better adsorption for low-concentration harmful substances than granular and pellet AC due to the excellent micropores on its surface [22,26].

In this study, the HC adsorption characteristics of AC (granular and pellet) and ACF were investigated to remove the evaporation gas of an automobile. The ACF was fabricated using various H_2_O activation times for pitch fibers to observe the relationship between the adsorption capacity of low-concentration evaporation gas and the pore structure of the ACF. The pore development mechanism of ACF was confirmed through the pore characteristics and crystal structure. The HC adsorption characteristics of AC and ACF were analyzed using various concentrations of n-butane according to the ASTM D5228 standard.

## 2. Experimental 

### 2.1. Preparation of Activated Carbon Fibers

ACF was fabricated from stabilized isotropic pitch fibers (GS Caltex, Jeonju, Korea). The precursor has a low ash content of less than 500 ppm and consists mostly of carbon. Three grams of pitch fiber was heated to 900 °C in nitrogen gas (500 mL/min of feeding rate) at a heating rate of 10 °C/min in a self-produced cylindrical steel tube furnace (diameter 130 mm × length 1000 mm) with SiC heaters, then activated with H_2_O (0.5 mL/min of feeding rate by a micro-feeder) for 20–60 min (holding or reaction time). The H_2_O was evaporated through a pre-heater heated at 200 °C and then introduced into a furnace. After activation, the flow of nitrogen was used to cool the furnace to room temperature to prepare the ACF. Each sample was named in the form ACF-(activation method)-(activation temperature)-(activation time).

The activation reaction of carbon crystallite with H_2_O is endothermic and takes the following stoichiometric form [27]:

(1) $$C+H_{2}O\to \mathrm{CO}+H_{2}\Delta H=+117\;\mathrm{kJ}/\mathrm{mol}$$

Commercial AC of the type used for canisters such as the BAX1500, BAXLBE (Pellet AC, Ingevity, North Charleston, SC, USA) and WVA1100 (Granular AC, Ingevity, North Charleston, SC, USA) were used for comparison with the ACF prepared in this work.

### 2.2. Characterizations

The pore characteristics of the ACF were analyzed using N_2_/77K adsorption–desorption isotherm curves by Bel-MAX (BEL Japan, Inc., Tokyo, JAPAN). The specific surface area of the ACF was calculated by the Brunauer–Emmett–Teller (BET) equation [28], and the pore size distribution curves of the ACF were measured by both a non-local density functional theory (NLDFT) [29,30] and the Barrett–Joyner–Jalenda (BJH) equation [31] according to the pore range. The crystallite structure of the ACF was analyzed using their X-ray diffraction (XRD, PANalytical, Almelo, The Netherlands) spectra. XRD patterns were measured using Cu Kα (1.542 A) in the range of 10–60° at a rate of 2°/min.

**Figure 1:**
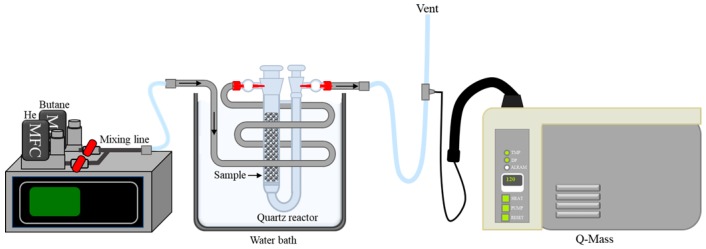
Schematic diagram of the butane working capacity test.

The butane working capacity (BWC) of the ACF was determined according to the ASTM D5228-16 standard [32]. The ACF was used to fill a U-shaped sample tube with a fixed volume of 16.7 mL. The sample tube was placed in a water bath maintained at 25 °C, and the butane was fed into the sample tube at 250 mL/min for 15 min. The mass change of the sample tube was measured using a balance, and the butane was adsorbed again for an additional 10 min. When a constant sample weight was determined, the sample tube was purged by helium gas at a rate of 300 mL/min for 40 min. BWC, butane activity, and butane retentivity were calculated using Equations (2)–(5). The change in the real-time butane concentration was measured using a quadrupole mass spectrometer (Q-mass) at the vent of the sample tube. A schematic diagram of the experimental setup is exhibited in Figure 1.


**Unit**

**Equation**
Butane working capacitorg/100 mL
$\frac{\left(D-E\right)}{\left(C-B\right)}\times A\times 100$
(2)Butane working capacitor%
$\frac{\left(D-E\right)}{\left(C-B\right)}\times 100$
(3)Butane activity%
$\frac{\left(D-C\right)}{\left(C-B\right)}\times 100$
(4)Butane retentivity%
$\frac{\left(E-C\right)}{\left(C-B\right)}\times 100$
(5)

where *A* is the apparent density, *B* is the weight of the sample tube and stoppers, *C* is the weight of the sample, sample tube, and stoppers; *D* is the weight of the saturated sample, sample tube, and stoppers; and *E* is the weight of the purged sample, sample tube, and stoppers.

## 3. Results and Discussion

**Figure 2:**
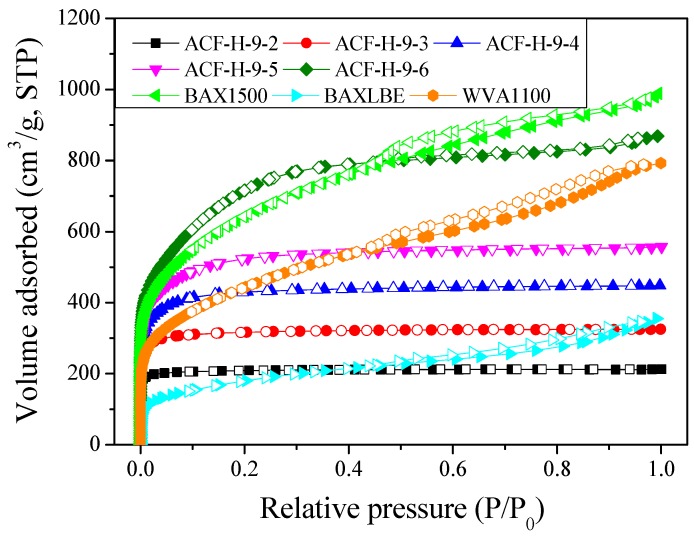
N_2_/77 K adsorption–desorption isotherm curves of pitch-based activated carbon fibers as a function of various H_2_O activation times and commercial activated carbons. ACF: activated carbon fibers.

Isothermal adsorption–desorption curves are the most powerful method for analyzing the pore characteristics of the ACF. Figure 2 exhibits the N_2_/77 K isothermal adsorption–desorption curves of the ACF. The curves of most ACF variants (ACF-H-9-2 to ACF-H-9-5) were classified as Type I by the IUPAC classification and were found to include mainly micropores [33]. Meanwhile, the curve of ACF-H-9-6 was classified as Type IV, and thus the volume ratio of mesopores to micropores was relatively high [33]. The hysteresis patterns were hardly observed in the curves of all the ACF variants, and only a few hysteresis patterns were observed in ACF-H-9-6. Therefore, it is considered that the pores of all the ACF are wedge-shaped, and that the pores are well-developed on the surface of the ACF. The adsorption–desorption isotherm curves of the AC (BAX1500, BAXLBE, and WVA1100) for canisters were determined to be Type IV, and very large hysteresis was observed. It is recognized that the AC has a high mesopore volume fraction within the total pore volume, and pot-shaped pores are present inside the pore structure.

Table 1 shows the textural properties of the ACF and AC. The specific surface area and total pore volume of the ACF increased from 840 to 2630 m^2^/g and from 0.33 to 1.34 cm^3^/g, respectively, with increased activation time. From ACF-H-9-2 to ACF-H-9-5, most of the porous structure was found to consist of micropores. As the activation time increased, the mesopore volume continuously increased. ACF-H-9-6 was observed to consist of about 37% of mesopores (of the total pore volume) and to have the highest specific surface area of all the ACF variants.

The AC for canisters has completely different pore structures compared to the ACF. The AC mesopores accounted for more than 57% of the total pore volume. The specific surface areas of the AC were in the order of BAX 1500 > WVA 1100 > BAX LBE. The pore ratio that ACF-H-9-6 exhibited was similar to that of the AC for canisters. ACF-H-9-6 had higher specific surface area and micropore volume than BAX 1500, but BAX 1500 had a higher mesopore volume than that of ACF-H-9-6.

**Figure 3:**
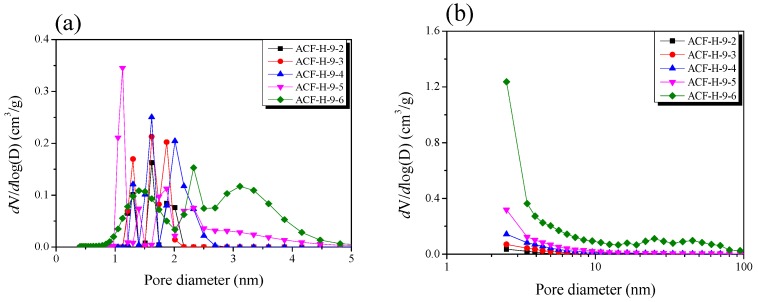
Pore size distribution of pitch-based activated carbon fiber as a function of various H_2_O activation time: (**a**) micropore size distribution by the nonlocalized density functional theory (NLDFT) method; (**b**) nesopore size distribution by the BJH equation.

Figure 3a exhibits the micropore size distribution curves of the ACF by an NLDFT method. The micropore size diameter was determined by gas sorption and estimated using nonlocalized density functional theory (NLDFT) and the grand canonical Monte Carlo method (GCMC) using the BELSORP evaluation software from the computer simulation. Figure 3a shows the typical pore size distribution of porous carbonaceous materials. The pore size distribution curves indicate that as the activation time increases to 40 min (ACF-H-9-4), the pore diameter increases and the width of the curve gradually increases. The pore size distribution curves of ACF-H-9-5 were observed to possess a narrow (~1 nm) and a broad (>2 nm) curve. ACF-H-9-6 had a broad curve, and the pore diameters increased from micropores to sub-mesopores. Therefore, it was confirmed that the mesopores were formed by the alteration of the micropores to mesopores by the widening of the pore diameters by further oxidation or by the collapse of the micropore walls. It was also confirmed that new micropore development continued in the variants up to ACF-H-9-6, as observed by the increase in micropore volume.

**Figure 4:**
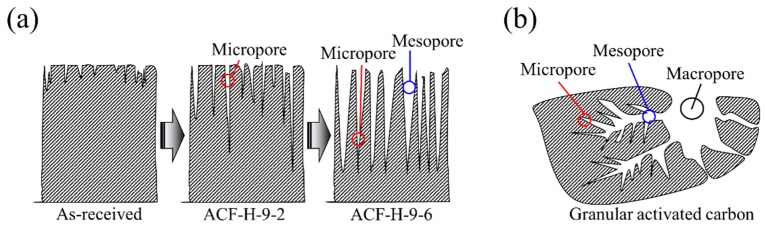
Schematic pore structure images of activated carbon fiber (**a**) and granular activated carbon (**b**).

Figure 3b shows the mesopore size distribution of the ACF calculated using the BJH equation. The mesopore volume of the ACF was found to increase with increasing activation time. ACF-H-9-6 had the most massive mesopore volume among the ACF variants. From ACF-H-9-2 to ACF-H-9-5, mesopores of less than 10 nm in pore diameter were mainly developed, whereas ACF-H-9-6 had mesopores of pore diameter <50 nm. Because hysteresis of the ACF is hardly observed in Figure 2 (which means that the pore shape is probably a wedge-shape, as shown in Figure 2), it can be inferred that the location micropores of the ACF gradually shift to deep inside as the activation time increases, while maintaining a wedge-shaped pore structure (Figure 4a). On the other hand, it is assumed that the AC has a pore structure of a unique jar shape, resulting in a high mesopore volume ratio and a large hysteresis curve (Figure 4b).

Physical activation is the process of oxidizing crystallites to form various pores. Therefore, the changes of crystallite and pore structure with increasing activation time are very closely related. XRD is an advantageous method for observing the crystallite structure of ACFs.

**Figure 5:**
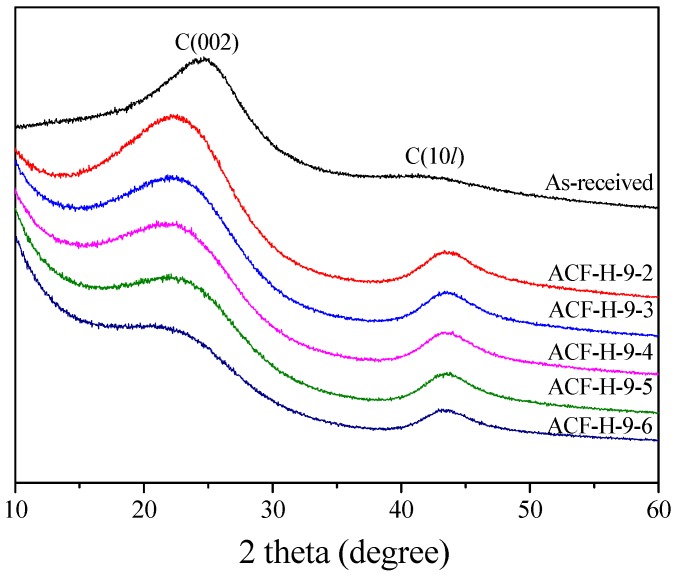
X-ray diffraction of pitch-based activated carbon fiber as a function of various H_2_O activation times.

In Figure 5, the XRD curve of the pitch fiber (as-received) exhibits a clear 002 peak and a well-developed domain characteristic of the inherent crystallite structure of the pitch. The XRD curves of the ACF exhibit the typical appearance of an isotropic carbon material. The 002 peak diffraction angle of the graphite crystallite is 26.56°, but the 002 peaks of the ACF samples are located at about 23°, indicating that the crystallite structures are considerably different from the structure of graphite. Besides this, the widely spread 10*l* peak indicates that each atomic layer is disordered and imperfectly laminated.

The XRD curves in Figure 3 were determined using the Bragg and Scherrer equations, and the interplanar spacings (d_002_ and d_10*l*_) and the crystallite sizes (L_c_ and L_a_) were measured. The structural parameters are shown in Table 2 and Figure 5.

**Figure 6:**
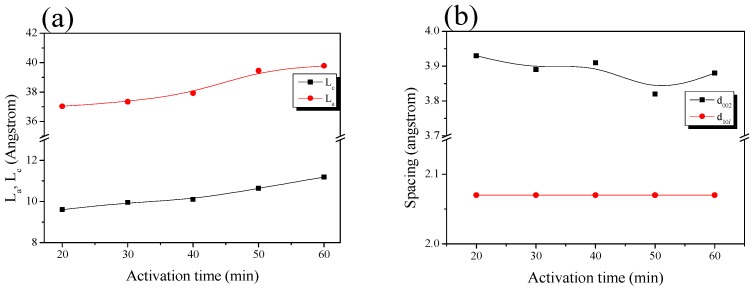
Structural characteristics of pitch-based activated carbon fibers as a function of various H_2_O activation conditions: (**a**) structural parameters; (**b**) interplanar distance.

In Figure 6a, the L_c_ (crystallite height) and L_a_ (crystallite size) of the ACF increases with increasing activation time. Generally, the XRD data of carbonaceous materials provides statistical data about the number of crystallite aggregates. It is known that amorphous and small crystallites are preferentially oxidized, compared to larger crystallites, during the activation process; that is, as the activation of a pitch fiber proceeds, amorphous parts and small crystallite are easily oxidized, which may appear to increase the relative size of the entire crystallite. Therefore, it is considered that L_c_ and L_a_ are increased by the oxidation of amorphous areas or small crystallites as the activation time increases. Moreover, a steady increase of L_c_ and L_a_ during long activation times seems to result in the sustained oxidation of amorphous regions or small crystallite. The low increase of L_a_ in ACF-H-9-6 is considered to be highly correlated with the increase of the mesopore volume (Table 2). The increase in the micropore volume is the result of the oxidation of amorphous or small crystallites, leading to an increase in the L_c_ and L_a_. By contrast, the increase in the mesopore volume is formed by the oxidation of the micropore walls (the edge of the large crystallite), resulting in a decrease in L_c_ and L_a_. Therefore, ACF-H-9-6 is considered to result in a tiny increase in L_a_ because this sample shows an increase in the micropore volume and a significant increase in the mesopore volume.

In Figure 6b, d_002_ decreases, and d_10*l*_ does not change significantly with increasing activation time. In the graphite crystal structure, the 002 planes are composed of strong hybridized sp^2^ bonds, and the vertical π bond on 002 planes displays weak interlayer bonding. The decrease of d_002_ is considered to be the result of the continuous oxidation of amorphous parts and small crystallites.

The butane working capacity (BWC) is a useful analytical method for evaluating the canister performance of ACF and AC. Table 3 lists the BWC, butane activity (BA), and butane retentivity (BR) of the ACF and AC measured according to ASTM D5228. The BA and BR indicate the butane adsorption capacity and the residual butane rate, respectively, after the desorption of the butane from the ACF. The BWC is defined as the difference between the butane adsorbed at saturation and that retained per unit volume of the ACF after a specified purge.

The BA and BR of the ACF increased with increasing activation time and were observed to range between 15.78–57.33% and 4.19–11.47%, respectively. The butane activity of the ACF was increased by the increase of the micropore and mesopore volume. It is generally known that AC with a high mesopore ratio can desorb the adsorbate more easily in the purging process. For example, as shown in Table 3, the BR of the AC for the canister was found to be lower with the increase in the mesopore volume ratio. However, the mesopore volume ratio of the ACF increased from ACF-H-9-3 to ACF-H-9-6, but the BR decreased. As shown in Figure 2, micropore development is more probable during pore development in the ACF. The micropores of the ACF were then transformed to mesopores, and new micropores developed deep inside the mesopores. Therefore, the micropores are uniformly formed on the surface of the ACF (up to ACF-H-9-5) and most of the pores are micropores. The ACF exhibited higher BA and BR values than those of AC with a similar specific surface area. The ACF is known to have faster adsorption characteristics than AC because of the formation of pores on its surface. This means that the ACF has a higher heat of adsorption than that of AC, resulting in desorption difficulty. Therefore, it is considered that ACF has better adsorption characteristics even if the specific surface area is similar to that of AC. On the other hand, because the AC has a higher mesopore volume ratio than the ACF, desorption can be easily performed during the purging process.

**Figure 7:**
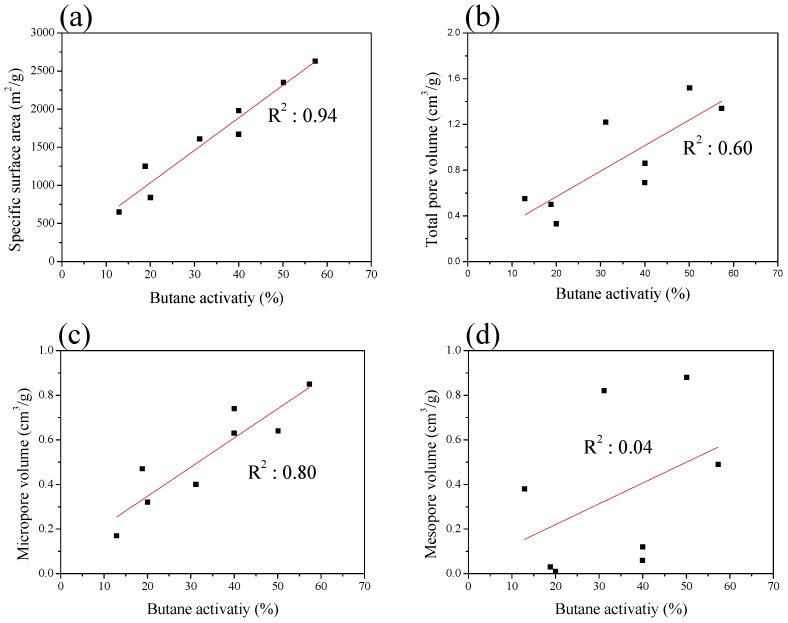
Correlations between the butane activity with specific surface area (**a**), total pore volume (**b**), micropore volume (**c**), and mesopore volume (**d**).

The BA of the AC was investigated by exploring its correlation with textural properties. In Figure 7, the BA of the ACF and AC appears to be linearly dependent on the specific surface area and microporosity. On the other hand, the BA was not found to be linearly related to the mesoporosity. These results are consistent with the results in Table 3 and suggest that the pore size may play an essential role in determining the BA of AC.

**Figure 8:**
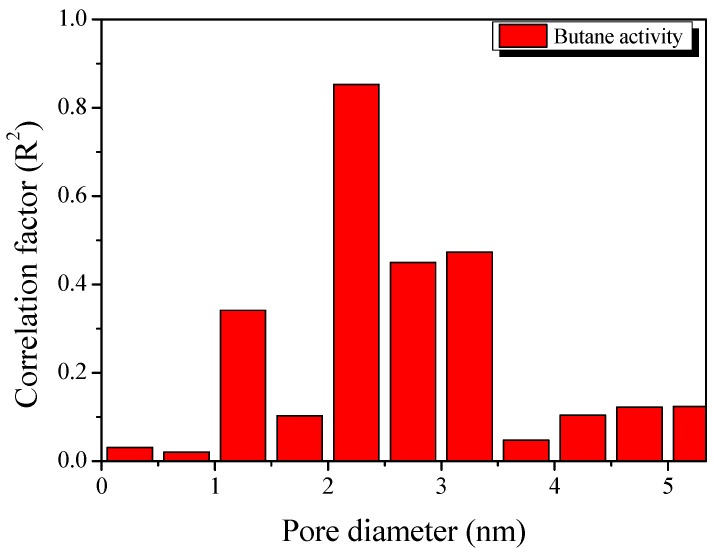
Correlations between the butane activity of activated carbon with pore volume. The *X*-axis exhibits the average pore size distribution after plotting the pore volume according to the pore diameter in 0.5 nm units using the average value of each pore size distribution.

Figure 8 exhibits the result of plotting the pore volume according to pore diameter in 0.5 nm units using the NLDFT method and then plotting the coefficient of determination with BA. It is considered that the BA of the ACF is determined by pores with diameters from 2.0 to 3.5 nm. Primarily, the BA was strongly dependent on pores with diameters from 2.0 to 2.5 nm. This result is consistent with those in previous studies [15] showing that mesopores (2.0–2.5 nm) are essential for providing the butane adsorption capacity.

**Figure 9:**
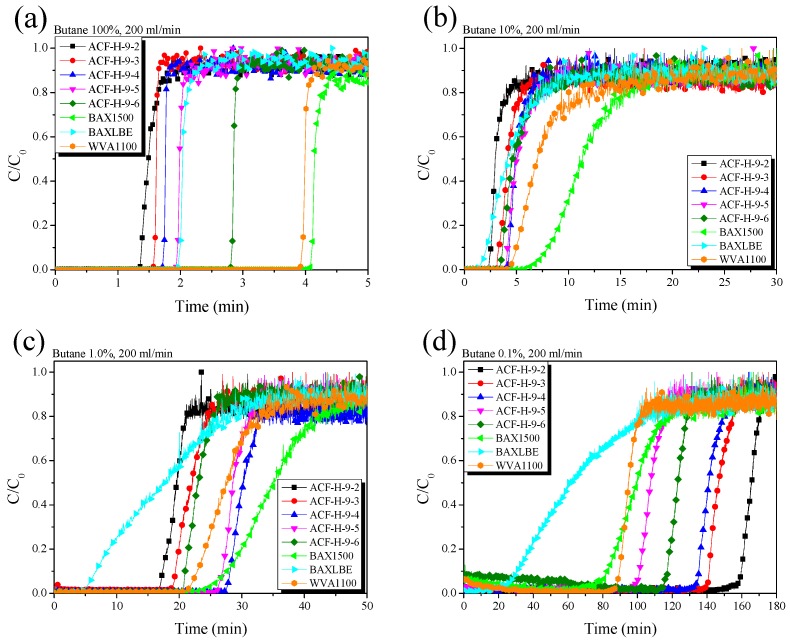
Breakthrough curves of pitch-based activated carbon fibers as a function of various H_2_O activation time and butane concentration: (**a**) 100% butane, (**b**) 10% butane, (**c**) 1.0% butane, and (**d**) 0.1% butane.

In order to meet the enhanced regulation of evaporation gas, it was necessary to develop an adsorbent with excellent adsorption capacity for HC at low concentrations. Because ASTM D5228 is used to evaluate the adsorption capacity of butane (100%) by mass change, it was impossible to conduct an experiment measuring butane at low concentration because the change in mass would be minimal. Figure 9 exhibits the change in concentration of the AC and ACF at various concentrations of butane using a Q-mass for the ASTM D5228 measurement.

As the butane concentration decreased, the slope of the curve near the breakthrough time of the AC decreased. On the other hand, the decrease in butane concentration did not significantly affect the curve slope of the ACF near the breakthrough time. In general, as the gas concentration decreases, the adsorption rate of the adsorbent decreases because the adsorption and the desorption reaction occur simultaneously.

The ACF has a faster adsorption rate than AC because micropores develop on the surface due to its inherent pore structure characteristics. Therefore, the adsorption rate of AC decreased as the concentration of butane decreased, suggesting that the slope of the curve decreased. On the other hand, because the adsorption rate of the ACF is faster than that of AC, it is considered that a change of the curved ACF slope does not appear, even if the concentration of butane decreases.

Figure 9a exhibits the change in concentration caused by the adsorption of 100% butane by the AC and ACF. In Table 3, the butane activity of the ACF is higher than that of AC. However, in Figure 9a, it can be seen that the breakthrough times of AC are longer than those of ACF. The apparent density of AC is about 1.9–2.3 times higher than that of ACF, so the mass of AC is higher, given the same volume. The butane adsorption capacity of the ACF was found to be proportional to the pore characteristics. In particular, ACF-H-9-6 exhibited the highest adsorption performance in the 100% butane-adsorption test because it had the highest specific surface area and highest total pore volume among the ACF variants.

Figure 9b shows the change in concentration caused by the adsorption of 10% butane by AC and ACF. In Figure 5b, the AC exhibits better butane adsorption characteristics than the ACF (Figure 5a), but the breakthrough times between the AC and ACF are very close. The breakthrough times of the ACF variants were almost the same, except for ACF-H-9-2. This result is attributed to the decrease in the adsorption rate of the ACF and AC due to the lower butane concentration.

Figure 9c shows the change in concentration caused by the adsorption of 1.0% butane by the AC and ACF. The slope of the curve near the breakthrough time of the AC was further reduced, while the curve of the ACF maintained a high slope. Moreover, ACF-H-9-4 and ACF-H-9-5 show breakthrough times similar to those of the AC. These results indicate that the adsorption rate of the AC is significantly lower than that of the ACF at very low butane concentrations. Among the ACF variants, although ACF-H-9-4 had a lower specific surface area and total pore volume than those of ACF-H-9-5 and ACF-H-9-6, it exhibited the best adsorption capacity for 1.0% butane. As shown in Figure 4, it can be recognized that ACF-H-9-4 may have micropores which formed mostly on the surface. In conclusion, ACF-H-9-4 exhibited excellent adsorption characteristics for 1.0% butane due to its well-developed micropore structure.

Figure 9d exhibits the change in concentration caused by the adsorption of 0.1% butane by AC and ACF. At this butane concentration, the ACF exhibited better adsorption characteristics than the AC. Moreover, the shorter the activation time of the ACF, the better the adsorption capacity observed. In Figure 2, the isothermal curves of the ACF prepared in this work showed Type 1 curves, and the hysteresis of the samples was negligible. This could mean that the pore shapes of the samples might be wedge-shaped or cylindrical slit-shaped. Moreover, as the activation time increases, the diameter of the pore inlet located on the surface of the ACF increases gradually. Therefore, as the activation time increases, the positions of pores with diameters of 2.0–2.5 nm, which affect the butane adsorption, are expected to gradually shift to the inside of the pore structure, meaning that the adsorption rate decreases.

## 4. Conclusions

In this study, the HC adsorption characteristics of AC and ACF were studied according to their pore structures. The ACF was fabricated using various H_2_O conditions to activate stabilized isotropic pitch fibers. From the results, the specific surface area and total pore volume of the ACF were determined to be 840–2630 m^2^/g and 0.33–1.34 cm^3^/g, respectively. A close relationship between BA and specific surface area was determined. It is also inferred that the sub-mesopore volume fraction in the range 2.0–2.5 nm primarily controls n-butane adsorption. The ACF exhibited higher butane activity than the AC, but the adsorption capacity was lower than AC for butane at a concentration of 10–100% due to the low apparent density. However, at a butane concentration of 0.1–1.0%, the intrinsic pore characteristics of the ACF were observed to have excellent adsorption capacity. With butane at 0.1% concentration, a higher butane adsorption capacity was observed with a lower specific surface area of the ACF.

Under the enhanced environmental regulations for automobile exhaust gas, an effective adsorbent is required for unburned-HC removal systems (canisters and ACS) with an excellent butane adsorption capability and a perfect adsorption property of butane at low concentrations. The results of this study show that AC exhibits a good butane adsorption capacity at high concentrations, and that ACF has an adsorption characteristic perfect for low concentrations of butane. Therefore, it is expected that an HC removal system incorporating AC with ACF would exhibit excellent performance without external accessories such as a honeycomb device.

## Figures and Tables

**Table 1:** Textural properties of pitch-based activated carbon fibers as a function of various H_2_O activation conditions and commercial activated carbon.

Sample	S_BET_^1^ (m^2^/g)	V_Total_^2^ (cm^3^/g)	V_Micro_^3^ (cm^3^/g)	V_Meso_^4^ (cm^3^/g)	Mesopore ^5^ Ratio (%)	Yield ^6^ (%)
ACF-H-9-2	840	0.33	0.32	0.01	3.0	55
ACF-H-9-3	1250	0.50	0.47	0.03	6.0	45
ACF-H-9-4	1670	0.69	0.63	0.06	8.7	38
ACF-H-9-5	1980	0.86	0.74	0.12	14.0	26
ACF-H-9-6	2630	1.34	0.85	0.49	36.6	15
BAX1500	2350	1.52	0.64	0.88	57.9	-
BAXLBE	650	0.55	0.17	0.38	69.1	-
WVA1100	1610	1.22	0.40	0.82	67.2	-

^1^ S_BET_: Specific surface area; Brunauer–Emmett–Teller (BET) method; $\frac{P}{v\left(P_{0}-P\right)}=\frac{1}{v_{m}c}+\frac{c-1}{v_{m}c}\cdot \frac{P}{P_{0}}$. ^2^ V_Total_: Total pore volume; BET method. ^3^ V_Meso_: Mesopore volume; Barrett–Joyner–Halender (BJH) method: $r_{p}=r_{k}+t$. (*r_p_* = actual radius of the pore, *r_k_* = Kelvin radius of the pore, *t* = thickness of the adsorbed film). ^4^ V_Micro_: Micropore volume; V_Total_-V_Meso_. ^5^ Mesorpore ratio: $\frac{V_{\mathrm{Meso}}}{V_{\mathrm{Total}}}\times 100$. ^6^ Yield: $\frac{\mathrm{Weight}\;\mathrm{of}\;\mathrm{activated}\;\mathrm{sample}}{\mathrm{Weight}\;\mathrm{of}\;\mathrm{carbonized}\;\mathrm{sample}\;\mathrm{input}}\times 100$.

**Table 2:** Structural parameters of pitch-based activated carbon fibers as function of various H_2_O activation conditions. FWHM: full width half maximum.

Sample	002 Peak				10*l* Peak			
	2θ (°)	d_002_ (Å)	FWHM (2θ)	L_c_ (Å)	2θ (°)	d_10*l*_ (Å)	FWHM (2θ)	L_a_ (Å)
ACF-H-9-2	22.62	3.93	8.44	9.61	43.78	2.07	4.73	37.03
ACF-H-9-3	22.83	3.89	8.16	9.95	43.79	2.07	4.69	37.34
ACF-H-9-4	22.73	3.91	8.03	10.10	43.75	2.07	4.62	37.92
ACF-H-9-5	23.25	3.82	7.63	10.64	43.76	2.07	4.44	39.46
ACF-H-9-6	22.92	3.88	7.25	11.19	43.69	2.07	4.40	39.78

**Table 3:** Butane working capacity of pitch-based activated carbon fibers as function of various H_2_O activation conditions.

Sample	Density (g/mL)	BWC ^1^ (g/100 mL)	BWC (%)	BA ^2^ (%)	BR ^3^ (%)
ACF-H-9-2	0.15	1.59	10.63	20.03	5.41
ACF-H-9-3	0.14	2.18	15.56	18.83	3.27
ACF-H-9-4	0.14	3.60	25.69	39.97	9.19
ACF-H-9-5	0.13	4.01	30.83	40.01	9.18
ACF-H-9-6	0.13	6.00	46.13	57.33	11.47
BAX 1500	0.31	15.03	50.11	50.11	7.43
BAX LBE	0.39	5.03	12.91	12.91	2.41
WVA 1100	0.28	8.73	31.17	31.17	5.87

^1^ BWC: butane working capacity. ^2^ BA: butane activity. ^3^ BR: butane retentivity.

## References

[1] Council of the European Union (CEU) (2015). Annex to the Commission Regulation Amending Regulation (EC) No 692/2008 as Regards Emissions from Light Passenger and Commercial Vehicles (Euro 6).

[2] California Air Resources Board Amendments to the Low-Emission Vehicle Program—LEV III. http://www.arb.ca.gov/msprog/levprog/leviii/leviii.htm.

[3] Manetas C., Sharifian L., Alexiadou P., Lafossas F.A., Mohammadi A., Koltsakis G. (2019). Modeling of the Competitive Storage of NOx and Sulfur in Automotive Exhaust Catalysts. Top. Catal..

[4] Kim H.G., Yu Y., Yang X., Ryu S.K. (2019). Carbon dioxide (CO_2_) concentrations and activated carbon fiber filters in passenger vehicles in urban areas of Jeonju, Korea. Carbon Lett..

[5] Saliba G., Saleh R., Zhao Y., Presto A.A., Lambe A.T., Frodin B., Sardar S., Maldonado H., Maddox C., May A.A. (2017). Comparison of Gasoline Direct-Injection (GDI) and Port Fuel Injection (PFI) Vehicle Emissions: Emission Certification Standards, Cold-Start, Secondary Organic Aerosol Formation Potential, and Potential Climate Impacts. Environ. Sci. Technol..

[6] Yue T., Yue X., Chai F., Hu J., Lai Y., He L., Zhu R. (2017). Characteristics of volatile organic compounds (VOCs) from the evaporative emissions of modern passenger cars. Atmos. Environ..

[7] Faith W.L., Goodwin J.T., Morriss F.V., Bolze C.J. (1957). Automobile Exhaust and Somog Formation. Air Pollut. Control Assoc..

[8] Kotin P., Falk H.L. (1963). Atmospheric Factors in Pathogenesis of Lung Cancer. Adv. Cancer Res..

[9] United States Environmental Protection Agency (EPA) (2014). Evaporative Emissions from On-Road Vehicles in MOVES2014.

[10] Hiltzik L.H., Jagiello J.Z., Tolles E.D., Williams R.S. (2003). Method for Reducing Emissions from Evaporative Emissions Control Systems. U.S. Patent.

[11] Ishikawa K., Abe S., Ishimura S. (2010). Activated Carbon Product in Sheet form and Element of Device for Preventing Transpiration of Fuel Vapor. U.S. Patent.

[12] Moyer D., Khami R., Bellis A., Luley T. (2017). Evolution of Engine Air Induction System Hydrocarbon Traps.

[13] Jamrog J.R., Pierce M.A., Johnson P.J. (2001). Evaporative Emission Canister for an Automotive Vehicle. U.S. Patent.

[14] Vaishnav D., Ehteshami M., Collins V., Ali S., Gregory A., Werner M. (2017). CFD Driven Parametric Design of Air-Air Jet Pump for Automotive Carbon Canister Purging. SAE Int. J. Passeng. Cars-Mech. Syst..

[15] Lee H.M., Baek J., An K.H., Park S.J., Park Y.K., Kim B.J. (2019). Effects of Pore Structure on n-Butane Adsorption Characteristics of Polymer-Based Activated Carbon. Ind. Eng. Chem. Res..

[16] Tolles E.D., Dimitri M.S., Matthews C.C. (1993). High Activity, High Density Activated Carbon. U.S. Patent.

[17] Brown T.C. (2017). Adsorption Properties from Pressure-Varying Langmuir Parameters: N-Butane and Isobutane on Activated Carbon. Energy Fuels.

[18] Yuan H., Jin B., Meng L.Y. (2018). Effect of the SBA-15 template and KOH activation method on CO_2_ adsorption by N-doped polypyrrole-based porous carbons. Carbon Lett..

[19] Jeguirim M., Belhachemi M., Limousy L., Bennicim S. (2018). Adsorption/reduction of nitrogen dioxide on activated carbons: Textural properties versus surface chemistry—A review. Chem. Eng. J..

[20] Kim M.J., Lee S., Lee K.M., Jo H., Choi S.S., Lee Y.S. (2018). Effect of CuO introduced on activated carbon fibers formed by electroless plating on the NO gas sensing. J. Ind. Eng. Chem..

[21] Ohc J.Y., You Y.W., Park J., Hong J.S., Heo I., Lee C.H., Suh J.K. (2019). Adsorption characteristics of benzene on resin-based activated carbon under humid conditions. J. Ind. Eng. Chem..

[22] Das D., Gaur V., Verma N. (2004). Removal of volatile organic compound by activated carbon fiber. Carbon.

[23] Miura K., Nakagawa H., Okamoto H. (2000). Production of high density activated carbon fiber by a hot briquetting method. Carbon.

[24] McCue J.C., Repik A.J., Miller C.E. (1987). Shaped Wood-Based Active Carbon. U.S. Patent.

[25] Happel J. (1949). Pressure Drop Due to Vapor Flow through Moving Beds. Ind. Eng. Chem..

[26] Zhang X., Gao B., Creamer A.E., Cao C., Li Y. (2017). Adsorption of VOCs onto engineered carbon materials: A review. J. Hazard. Mater..

[27] Wigmans T. (1989). Industrial aspects of production and use of activated carbons. Carbon.

[28] Brunauer S., Emmet P.H., Teller W.E. (1938). Adsorption of gases in multimolecular layers. J. Am. Chem. Soc..

[29] Lastoskie C., Gubbins K.E., Quirke N. (1993). Pore size distribution analysis of microporous carbons: A density functional theory approach. J. Phys. Chem..

[30] Olivier J.P., Conklin W.B., Szombathely M.V. (1994). Determination of pore size distribution from density functional theory: A comparison of nitrogen and argon results. Stud. Surf. Sci. Catal..

[31] Barrett E.P., Joyner L.G., Halenda P.P. (1951). The determination of pore volume and area distributions in porous substances. I. Computations from nitrogen isotherms. J. Am. Chem. Soc..

[32] American Society for Testing and Materials (1992). Standard Test Method for Determination of Butane Working Capacity of Activated Carbon.

[33] Sing K.S.W., Everett D.H., Haul R.A.W., Moscou L., Pierotti R.A., Rouquerol J., Siemieniewska T. (1985). Reporting physisorption data for gas/solid systems with special reference to the determination of surface area and porosity. Pure Appl. Chem..

